# Methods of skeletal age assessment using hand and wrist radiographs in children and their applicability across different ethnicities: systematic review and meta-analysis protocol

**DOI:** 10.1186/s13643-025-03030-8

**Published:** 2026-05-21

**Authors:** Sameera Wijayawardhana, Deepan Jayapala, Cree Gaskin, Jerrard Fernando, Yasith Mathagasinghe, Pujitha Wickramasinghe

**Affiliations:** 1 Department of Anatomy, Faculty of Medicine, University of Kelaniya, Ragama, Sri Lanka; 2 Department of Anatomy, Faculty of Medicine, University of Moratuwa, Moratuwa, Sri Lanka; 3 Department of Radiology and Medical Imaging, University of Virginia, Charlottesville, USA; 4 Lady Ridgway Hospital for Children, Colombo, Sri Lanka; 5 Centre for Human Anatomy Education, Department of Anatomy and Developmental Biology, School of Biomedical Sciences, Faculty of Medicine, Nursing and Health Sciences, Monash University, Melbourne, Australia; 6 Department of Paediatrics, Faculty of Medicine, University of Colombo, Colombo, Sri Lanka

**Keywords:** Skeletal age, Bone age, Hand, Radiographs, X-ray

## Abstract

**Background:**

Skeletal age assessment using hand and wrist X-rays is commonly used to determine the biological maturity of children. It is invaluable when the chronological age is not known or in diagnosing and treating various endocrine, genetic, and developmental disorders as well as in a forensic context. Skeletal age is affected by both internal and external factors, leading to differences in skeletal maturity between children of different ethnicities. Regional studies have even questioned the applicability of current international skeletal age references across ethnicities.

**Methods:**

A comprehensive literature search will be conducted utilising the PubMed, Scopus, Web of Science, EMBASE, and Ovid online databases to identify existing publications. A combination of Medical Subject Headings (MeSH) terms and keywords, including “Skeletal age”, “bone age”, “radiograph”, and “hand”, will be used. Data screening and extraction will be done with Covidence software. Two independent reviewers will screen literature against predefined inclusion and exclusion criteria in abstract and full-text forms. A third reviewer will be consulted if consensus cannot be reached. The process will be reported in whole and presented in a PRISMA flow diagram. Study quality will be assessed by using the JBI critical appraisal tool. A meta-analysis will be conducted.

**Discussion:**

This review will present a comprehensive analysis of existing literature on the applicability of skeletal age assessment methods using hand and wrist radiographs across different ethnicities. Further, this will be highlighting both strengths and gaps in existing knowledge. The inclusion of high-quality data will facilitate a quantitative meta-analysis for the exploration of ethnic differences in skeletal age assessments, ultimately laying the foundation for the development of population-specific reference standards.

**Systematic review registration:**

PROSPERO registration ID CRD42025646662.

**Supplementary Information:**

The online version contains supplementary material available at 10.1186/s13643-025-03030-8.

## Background

Skeletal age resembles the level of biological and structural maturity of a child’s skeletal system [1]. The skeletal system is a dynamic anatomical structure that undergoes growth and maturation, which is evident on radiography, making it a valuable tool for assessing a child’s growth and maturation. Beyond its clinical significance in diagnosing and managing various endocrine, genetic, and developmental disorders in children, analysis of skeletal age also contributes to various applications in forensic and anthropological contexts [2].

Skeletal age assessment based on the evaluation of epiphyseal ossification patterns and fusion of bones in the hand and wrist X-rays [3, 4] is recognised as a reliable indicator of skeletal maturity in children [2, 4]. Of the numerous methods of skeletal age assessment using hand radiographs, Greulich and Pyle (GP) atlas and Tanner-Whitehouse (TW) method are widely used reference standards in clinical practice [5]. The rate of ossification of an individual is affected by both extrinsic factors, such as environmental factors and socioeconomic status, and internal factors, such as genetics and illness [6, 7]. Hence, a reference standard for one population might not necessarily be applicable to another population. The commonly used hand and wrist-based skeletal age assessment methods have been developed using cohorts of Caucasian children, raising concerns about their reliability and applicability in diverse populations [8, 9]. In response, some countries have developed country-specific skeletal age reference standards [9, 10], but no universally accepted method applies across different ethnicities [5].

This systematic review and meta-analysis aim to analyse the applicability of skeletal age assessment methods using hand and wrist X-rays among different ethnicities. Further, we aim to identify the various methods related to their anatomical bases, clinical applications, and applicability in healthy children, as described in the existing literature. This review will consolidate the current knowledge and address limitations in existing methodologies. Identifying these methods will ultimately facilitate the development of ethnicity-specific skeletal age assessment standards.

## Methods/design

The proposed systematic review will be conducted complying with the Preferred Reporting Items for Systematic Reviews and Meta-Analyses (PRISMA) 2020 guidelines [10]. It will follow the recommended standards for conducting and reporting systematic reviews. We consulted the International Prospective Register of Systematic Reviews (PROSPERO) (https://www.crd.york.ac.uk/prospero/) to identify if there were ongoing protocols for systematic reviews similar to the present protocol. However, we found no relevant records as of February 2, 2024. This systematic review protocol is registered on PROSPERO and is reported according to the PRISMA-P 2015 checklist [11] (Supplementary File 1).

### Stage 1: identifying the research question

This review aims to explore the current literature pertaining to methods of skeletal age assessment using hand radiographs in healthy children. The primary objective of the review is to examine the methods of skeletal age assessment using hand and wrist radiographs in children and their applicability across different ethnicities. Our primary outcome is the applicability of skeletal age assessment methods, quantified as the mean difference (MD) between the assessed skeletal age and the chronological age. This allows for a direct measure of over- or under-estimation by a given method in a specific ethnic group.

The specific objectives of the systematic review are as follows:

- To describe the methods of skeletal age assessment using hand radiographs applied across different ethnic populations
- To analyse the extent of ethnic variation in skeletal age development using hand radiographs and its implications for developing population-specific reference standards
- To describe the anatomical and physiological basis of methods used for skeletal age assessment based on hand radiographs in healthy children

### Stage 2: search strategy

This systematic review will consider studies conducted prior to December 31, 2024. The review will include original descriptive observational studies, experimental studies, and randomised controlled trials. Full-text original studies conducted among healthy children will be included without any language restrictions. Studies with inadequate data on ethnicity and studies involving children with pathological conditions will be excluded. In case more than one manuscript is published within the same study, the manuscript with the highest amount of precise information will be used. Table 1 summarises the inclusion and exclusion criteria developed. The corresponding authors will be contacted in the event of inadequate information reported in the article. The criteria might be adjusted during the screening after consensus among the reviewers. Any of the adjustments will be applied to all studies accordingly.

**Table 1:** Inclusion and exclusion criteria

Component	Inclusion criteria	Exclusion criteria
Population (P)	Healthy children of age < 18 years	Children with pathological conditions affecting skeletal development. Specifically excluding chronic steroid use; deficiencies of hormones related to bone growth (growth hormone, thyroxine, parathyroid hormone); major nutritional deficiencies and vitamin D deficiency; pathological conditions of the bone (osteogenesis imperfecta, rickets, Paget’s disease); or syndromic children
Intervention (I)	Skeletal age assessment using hand and wrist radiographs	Studies have not used hand and wrist radiographs for skeletal age assessment
Comparison (C)	Not mandatory May include comparisons between different ethnicities or assessment methods	Not applicable
Outcome (O)	Method of skeletal age assessment and applicability of the technique across different ethnicities	Studies lacking data on skeletal age assessment methods or their applicability across ethnicities
Study design	Original descriptive observational studies, experimental studies, and randomised controlled trials	Reviews, editorials, commentaries, case reports, and studies with inadequate data on ethnicity
Language and other	There are no language restrictions	In cases of multiple publications from the same study, we will collate all available information from the multiple manuscripts

A comprehensive literature search will be conducted utilising the PubMed, Scopus, Web of Science, EMBASE, and Ovid online databases to identify existing publications. The search strategy will use all identified keywords and index terms. A combination of Medical Subject Headings (MeSH) terms and keywords will be used to search across all fields. Keywords include “skeletal age”, “bone age”, “X-ray”, “radiograph”, and “hand” connected with Boolean operators. Additionally, the “Human” filter will be used. The search strategy is in Supplementary File 2.

### Stage 3: study selection

The final search results for screening will be exported into Covidence for duplicate removal. Titles and abstracts will be screened by two independent reviewers who are familiar with objectives, definitions, and eligibility criteria. Any discrepancies will be resolved through discussion, and if a consensus cannot be reached, a third reviewer will be consulted. The data extraction sheets will be pilot-tested using five randomly selected studies. To refine the inclusion and exclusion criteria, an assessment will be conducted once 10% of the studies have been screened by both reviewers to determine whether redefining the requirements is necessary. Studies that are filtered through screening will be retrieved in full-text form. Two independent reviewers will read the full text to further filter it against the eligibility criteria, while any discrepancies will be resolved through discussion with a third reviewer. The process will be reported as a whole and presented in a PRISMA flow diagram.

The overall strength and certainty of the body of evidence for each outcome will be assessed using the GRADE (Grading of Recommendations Assessment, Development and Evaluation) approach. Study quality will be assessed by using JBI critical appraisal tools and heterogeneity will be assessed using *I*^2^. The evidence will be graded as high, moderate, low, or very low, based on considerations of study design, risk of bias (from the JBI assessments), inconsistency (heterogeneity), indirectness, imprecision, and publication bias [12].

### Stage 4: data extraction, analysis, and reporting

A minimum of two reviewers will conduct data extraction independently. The data extraction variables are listed under study characteristics, population characteristics, methodological characteristics, outcome, limitations, and recommendations (Supplementary file 3). The Covidence software will be further utilised for data extraction. Once the selection process is complete, the team will conduct a pilot data extraction to identify any necessary adjustments to the extraction template. The meta-analysis will be conducted using a metafor package in R. We will compute standardised mean differences (SMDs) or other relevant effect size measures to quantify the ethnic differences in skeletal age in each assigned sex at birth. A random-effects model will be employed to account for between-study variability, considering the potential heterogeneity among the included studies. Statistical heterogeneity between studies will be quantified using the *I*^2^ statistic and Tau^2^. We will investigate sources of heterogeneity through pre-specified subgroup analyses, testing significant differences between groups.

The subgroups will include as follows (Table 2).

**Table 2:** Subgroup categories

Subgroup category	Definition
Ethnicity	Caucasian, Asian, African, Hispanic ((if reported and defined by primary studies)
Sex	Male/female
Age group	Early childhood (0–4 years)/middle childhood (5–10 years)/puberty (11–15 years)/late adolescence (16–18 years)
Method of assessment	Greulich-Pyle (GP)/Tanner-Whitehouse (TW)/automated
Geographic distribution	Asia/Europe/North America/South America/Oceania
Socioeconomic status	High/middle/low (if reported)
Study design	Observational/experimental or RCT studies

Publication bias will be evaluated through visual inspection of funnel plots and formal tests such as Egger’s test. A primary analysis will be conducted without differential weighting by study design. A subgroup analysis by study design (observational vs. experimental/RCT) will be conducted to explore whether effect estimates differ systematically by design type. If significant heterogeneity (*I*^2^ > 75%) is attributable to study design, we will consider presenting the results of separate meta-analyses for observational and experimental/RCT studies.

Meta-regression: If sufficient data are available (typically >10 studies per covariate), we will conduct a random-effects meta-regression to explore the relationship between the effect size (mean difference in skeletal age) and continuous study-level variables, such as the mean chronological age of the sample or the year of publication.

Handling high heterogeneity: In the event of substantial statistical heterogeneity (e.g. *I*^2^ > 75%) that cannot be adequately explained by our planned subgroup analyses or meta-regression, we will not present a single pooled estimate. Instead, we will prioritise a narrative synthesis of the results, potentially supplemented with separate forest plots for key subgroups to visually present the divergent findings.

All studies, regardless of their assessed risk of bias, will be included in the primary meta-analysis to provide a comprehensive overview of the available literature. However, the influence of study quality will be formally investigated through a pre-planned sensitivity analysis, where we will repeat the meta-analysis after excluding studies deemed to have a high risk of bias. This will allow us to determine if the overall conclusions are robust or are being unduly influenced by studies with significant methodological limitations. Furthermore, the risk of bias for individual studies will be a key domain in our final GRADE assessment to determine the overall certainty of the evidence.

This review will use the following definitions:

- “Chronological age”—The time that has passed since the birth of an individual
- “Skeletal age”—The age expressed in years that corresponds to the level of maturation of bones
- “Methods of assessment”—A specific technique or procedure for determining a child’s skeletal age, typically by interpretation of hand radiographs compared against established reference standards
- “Hand X-rays”—Radiographic images of the hand and wrist, commonly used in skeletal age assessment since carpal bones show distinct and measurable changes, allowing for skeletal age assessment
- “Healthy children”—Children who are free from conditions or diseases that could impact normal bone growth and development, ensuring their growth reflects the typical growth pattern of the related population

## Discussion

This will be the first systematic review to evaluate the applicability of skeletal age assessment methods using hand and wrist radiographs across different ethnicities. By incorporating a wide range of study designs, including observational, experimental, and randomised controlled trials (RCTs), the review will provide a comprehensive analysis of existing evidence. The inclusion of high-quality data will facilitate a quantitative synthesis using meta-regression, which will allow for a robust exploration of ethnic differences in skeletal age assessments. By comparing various assessment methods, this review aims to determine their applicability across diverse populations, ultimately laying the foundation for the development of population-specific reference standards.

## Supplementary Information

Supplementary Material 1.

Supplementary Material 2.

Supplementary Material 3.

## Data Availability

Not applicable.

## Abbreviation

JBI: Joanna Biggs Institute
