# Supplementary material for: Methods of skeletal age assessment using hand and wrist radiographs in children and their applicability across different ethnicities: systematic review and meta-analysis protocol
Source: Syst Rev. 2026 May 21;15:204. doi: 10.1186/s13643-025-03030-8 (PMC13371705; doi:10.1186/s13643-025-03030-8)
Supplement: Supplementary file 2 — Supplementary Material 2. [file 13643_2025_3030_MOESM2_ESM.docx]

Supplementary file 1

**Search string**

(((age determination by skeleton[MeSH Terms]) OR ("skeletal age" OR "bone age"))

AND

((((xray[MeSH Terms]) OR (X-ray)) OR (radiograph)) OR (radiography)))

AND

(hand OR "upper limb" OR wrist OR capus OR carpal OR radius OR ulna OR ulnar OR radial OR scaphoid OR lumate OR triquetral OR pisiform OR trapezium OR trapezoid OR capitate OR hamate)

**Expanded search string with human filter**

(("age determination by skeleton"[MeSH Terms] OR ("skeletal age"[All Fields] OR "bone age"[All Fields])) AND ("x rays"[MeSH Terms] OR ("diagnostic imaging"[MeSH Subheading] OR ("diagnostic"[All Fields] AND "imaging"[All Fields]) OR "diagnostic imaging"[All Fields] OR "x ray"[All Fields] OR "x rays"[MeSH Terms] OR "x rays"[All Fields]) OR ("radiograph"[All Fields] OR "radiographed"[All Fields] OR "radiographer"[All Fields] OR "radiographer s"[All Fields] OR "radiographers"[All Fields] OR "radiographic"[All Fields] OR "radiographical"[All Fields] OR "radiographically"[All Fields] OR "radiographics"[All Fields] OR "radiographing"[All Fields] OR "radiographs"[All Fields]) OR ("diagnostic imaging"[MeSH Subheading] OR ("diagnostic"[All Fields] AND "imaging"[All Fields]) OR "diagnostic imaging"[All Fields] OR "radiography"[All Fields] OR "radiography"[MeSH Terms] OR "radiographies"[All Fields] OR "radiographys"[All Fields])) AND ("hand"[MeSH Terms] OR "hand"[All Fields] OR "upper limb"[All Fields] OR ("wrist"[MeSH Terms] OR "wrist"[All Fields] OR "wrist joint"[MeSH Terms] OR ("wrist"[All Fields] AND "joint"[All Fields]) OR "wrist joint"[All Fields] OR "wrists"[All Fields] OR "wrist s"[All Fields]) OR "capus"[All Fields] OR ("carpals"[All Fields] OR "wrist joint"[MeSH Terms] OR ("wrist"[All Fields] AND "joint"[All Fields]) OR "wrist joint"[All Fields] OR "carpal"[All Fields] OR "carpal bones"[MeSH Terms] OR ("carpal"[All Fields] AND "bones"[All Fields]) OR "carpal bones"[All Fields]) OR ("radius"[MeSH Terms] OR "radius"[All Fields]) OR ("ulna"[MeSH Terms] OR "ulna"[All Fields]) OR "ulnar"[All Fields] OR ("radial"[All Fields] OR "radially"[All Fields] OR "radials"[All Fields]) OR ("scaphoid bone"[MeSH Terms] OR ("scaphoid"[All Fields] AND "bone"[All Fields]) OR "scaphoid bone"[All Fields] OR "scaphoid"[All Fields] OR "scaphoid s"[All Fields] OR "scaphoids"[All Fields]) OR "lumate"[All Fields] OR ("triquetrum bone"[MeSH Terms] OR ("triquetrum"[All Fields] AND "bone"[All Fields]) OR "triquetrum bone"[All Fields] OR "triquetral"[All Fields]) OR ("pisiform bone"[MeSH Terms] OR ("pisiform"[All Fields] AND "bone"[All Fields]) OR "pisiform bone"[All Fields] OR "pisiform"[All Fields] OR "pisiforme"[All Fields]) OR ("trapezium bone"[MeSH Terms] OR ("trapezium"[All Fields] AND "bone"[All Fields]) OR "trapezium bone"[All Fields] OR "trapezium"[All Fields] OR "trapeziums"[All Fields]) OR ("trapezoid bone"[MeSH Terms] OR ("trapezoid"[All Fields] AND "bone"[All Fields]) OR "trapezoid bone"[All Fields] OR "trapezoid"[All Fields] OR "trapezoidal"[All Fields] OR "trapezoidally"[All Fields] OR "trapezoids"[All Fields]) OR ("capitate bone"[MeSH Terms] OR ("capitate"[All Fields] AND "bone"[All Fields]) OR "capitate bone"[All Fields] OR "capitate"[All Fields] OR "capitates"[All Fields]) OR ("hamate bone"[MeSH Terms] OR ("hamate"[All Fields] AND "bone"[All Fields]) OR "hamate bone"[All Fields] OR "hamate"[All Fields] OR "hamates"[All Fields]))) AND (humans[Filter])
