# Supplementary material for: Methods of skeletal age assessment using hand and wrist radiographs in children and their applicability across different ethnicities: systematic review and meta-analysis protocol
Source: Syst Rev. 2026 May 21;15:204. doi: 10.1186/s13643-025-03030-8 (PMC13371705; doi:10.1186/s13643-025-03030-8)
Supplement: Supplementary file 3 — Supplementary Material 3. [file 13643_2025_3030_MOESM3_ESM.docx]

Supplementary file 3

Variables to extract

1. **Study characteristics**
2. First author
3. Title of the study
4. Year of publication
5. Study design
6. **Population characteristics**
7. Sample size
8. National origin / Region/ country of study
9. Ancestry
10. Ethnicity of the population
11. Age range of the population
12. Assigned sex at birth distribution of the population
13. **Methodological characteristics**
14. Method of skeletal age assessment (GP/TW/ Automated)
15. Reference standard used
16. Anatomical and physiological basis of the method
17. X ray acquisition method ( From hospital database/ Xrays taken for study purpose)
18. **Outcome**
19. Chronological age – Mean and standard deviation among age groups and genders
20. Skeletal age - Mean and standard deviation among age groups and sexes
21. Mean difference between chronological and skeletal age
22. Clinical Agreement Rate ( The proportion of skeletal age assessments falling within ±1 year of the chronological age )
23. Reliability metrics – intra- and inter-rater variability
24. Heterogeneity metrics across subgroups – to be calculated
25. Bias observed
26. **Limitations and recommendations**
27. Limitations of the study
28. Recommendations
